# Notes

**Published:** 1900-08

**Authors:** 


### NOTES.

Veterinarians of the far West who have become accustomed to rely on the merits of the drugs and chemicals furnished by Merck & Co., of New York, will now be better served by the establishment of a branch house in Chicago, where all their products will be accessible.

A recent *Bulletin* of the Nebraska Experiment Station, referring to the danger of feeding sorghum, calls special attention to the fact that what danger exists is rather transitory in the sorghum, and due to its being fed at times when certain changes have taken place due to unhealthy growth of the plant.

Veterinarian Shaw, of Benson, Arizona, writes of the welcome reception of the Journal in that far-away locality, where the hand of a fellow-practitioner has not been felt for nearly six months. In addition to quarantine-line work and the importation of animals from Mexico, Dr. Shaw has also the inspection of sheep entering into inter-State trade. The season in that district has been very dry, causing heavy losses among the animals.

